# An innovative approach for the evaluation of prolonged disorders of consciousness using NF-L and GFAP biomarkers: a pivotal study

**DOI:** 10.1038/s41598-022-21930-w

**Published:** 2022-11-02

**Authors:** L. Coppola, P. Mirabelli, D. Baldi, G. Smaldone, A. Estraneo, A. Soddu, A. M. Grimaldi, G. Mele, Marco Salvatore, Carlo Cavaliere

**Affiliations:** 1 IRCCS Synlab SDN, Napoli, Italy; 2 Istituto Di Ricovero E Cura a Carattere Scientifico (IRCCS) Fondazione Don Carlo Gnocchi, Florence, Italy; 3 Department of Physics and Astronomy, Western Institute of Neuroscience, University of Western Ontario, London, ON Canada

**Keywords:** Neuroscience, Biomarkers, Neurological disorders

## Abstract

Behavioral assessments during the clinical evaluation in prolonged disorders of consciousness patients could be not sufficient for a correct diagnosis and prognostication. To this aim, we used an innovative approach, involving the ultra-sensitive determination of biological markers, correlating them with imaging parameters to investigate the prolonged disorders of consciousness (pDoC).We assessed the serum concentration of neurofilament light chain(NF-L) and glial fibrillary acidic protein (GFAP) in pDoC (n = 16), and healthy controls (HC, n = 6) as well as several clinical imaging parameters such as Fractional Anisotropy (FA), Whole Brain SUV, and White Matter Hyperintensities volumes (WMH) using PET-MRI acquisition. As for differential diagnosis task, only the imaging WMH volume was able to discriminate between vegetative state/unresponsive wakefulness syndrome (VS/UWS), and minimally conscious state (MCS) patients (*p*-value < 0.01), while all selected markers (both imaging and in vitro) were able to differentiate between pDoC patients and HC. At subject level, serum NF-L concentrations significantly differ according to clinical progression and consciousness recovery (*p*-value < 0.01), highlighting a potential play for the longitudinal management of these patients.

## Introduction

### Prolonged disorders of consciousness patients

Patients with prolonged disorders of consciousness (pDoC) following severe brain injury might show important issues related to diagnosis of the consciousness level^1^. Moreover, disentangling patients in the vegetative state/unresponsive wakefulness syndrome (VS/UWS, awake but unaware patients) from patients in the minimally conscious state (MCS, patients with minimal but reproducible behavioral signs of consciousness) or emerged from the minimally conscious state (EMCS) is critical for predicting clinical outcomes, as patients in the MCS have a higher probability to recover full consciousness, with respect to patients in VS/UWS^2,3^. To improve diagnostic accuracy, at least five repeated behavioral assessments by means of validated assessment tools such as the Coma Recovery Scale-Revised (CRS-R ^4^) have been strongly recommended in the recent AAN and EAN guidelines on patients with pDoC^1,5^. However, clinical signs of consciousness might be hampered by arousal fluctuation even on the same day and possible co-existing motor language, or covert cognition^6,7^, and only advanced assessment tools (i.e., functional neuroimaging or neurophysiology) can reveal patients with covert cognition ^8^.

### Innovative markers in pDoC

Innovative neuroimaging methods, such as integrated MRI, EEG and/or ^18^F FDG-PET approaches can recognize residual neural activity and functional connectivity independently from their abilities to produce intentional behaviors ^9,10^. However, advanced neuroimaging cannot be frequently applied, or when possible, not applied in a large number of patients due to technical issues in acquisition and analysis^11,12^. In this scenario, a multidisciplinary approach, combining data obtained from clinical evaluation and neuroimaging could help clinicians to improve diagnosis and follow the clinical outcome with more data available for pDoC patients ^13,14^. Furthermore, the study and the introduction of new peripheral blood biomarkers are pivotal in the diagnosis of the pDoC and for the neurological outcome’s prediction after severe brain injury.

In particular, the Neurofilament light chain (NF-L) marker^15^, a 68 kDa cytoskeletal neuron-specific protein, is a biomarker for acute and post-acute brain conditions following a severe brain injury caused by an anoxic, hemorrhagic, or traumatic event ^16,17^. After the neuronal injury, serum NF-L levels rise rapidly and remain elevated for prolonged periods even 1 year after trauma^18,19^. It represents a highly sensitive biochemical biomarker of neuron decay, measurable directly from serum or plasma, increasing patient compliance with potential use for repeated monitoring during the evolution of the disease. In addition to the study of NF-L for the evaluation of brain injury, glial markers are useful for the evaluation of the recovery from the neurodegeneration process. The Glial fibrillary acidic protein (GFAP), a brain-specific marker of astrogliosis was deepened, representing a monomeric intermediate filament protein of the astrocytes^20,21^. The present retrospective study aimed at investigating the possible diagnostic and/or prognostic role of innovative blood markers analyzed and clinical imaging parameters in patients with pDoC. For these purposes we evaluated the serum NF-L e GFAP markers in pDoC patients, correlating them with clinical assessment and imaging data such as, fractional anisotropy (FA), brain Standardized Uptake Value (SUV), brain volume lesion mm^3^ and White Matter Hyperintensities (WMH), and investigating their trend in a longitudinal single-subject framework.

## Results

### Demographic and clinical characteristics

**Figure 1:**
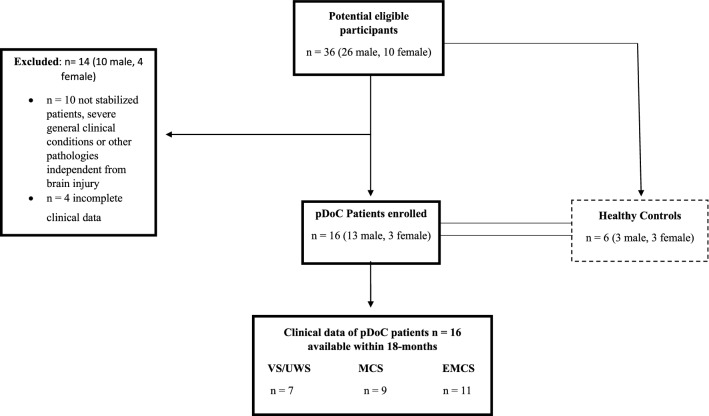
Flow chart of the retrospective study and patients' levels of consciousness post cerebral injury. VS/UWS, Vegetative State/Unresponsive Wakefulness Syndrome; MCS, Minimally Conscious State; EMCS, Emergence from a Minimally Conscious State refers to pDoC patients who recovered, re-staged and included in the data analyses for the evaluation of the markers.

The selection criteria of the patients included in this study are presented in Fig. 1. A total of 16 pDoC patients {VS/UWS = 7, time from injury (TFI) mean = 7 months, range [3–14 months], MCS patients = 9, TFI mean = 5 months range [1–14 months]}and 6 HC were included for NF-L and GFAP dosage as well as imaging analysis. Table 1 reports the demographic and clinical characteristics of the cohort analyzed. Moreover, 5 out of 16 pDoC patients were also evaluated during the follow-up at T1 (1–6 months), T2 (7–12 months), and T3 (13–18 months) post brain injury.

**Table 1:** Patient characteristics.

Retrospective study n = 22	
Disorders of consciousness patients	n = 16
Age (years), mean	45 (18–73)
Male n (%)	13 (81%)
Etiology (n)	
Traumatic	3
Anoxic	6
Vascular	7
Lesion volume mean (mm^3^)	
Traumatic	14,256
Anoxic	443
Vascular	19,946
pDoC state (n) at T1	
VS/UWS	7
MCS	9
TFI (months) at T1	
VS/UWS	mean = 5; range [3–14 months]
MCS	mean = 7; range [1–14 months]
Healthy controls	n = 6
Age (years), mean	37 (29–63)
Male n (%)	3 (50%)

Report of the demographic and etiology characteristics of the cohort analyzed (n = 22); TFI = Time from Injury.

### pDoC markers in diagnosis

**Figure 2:**
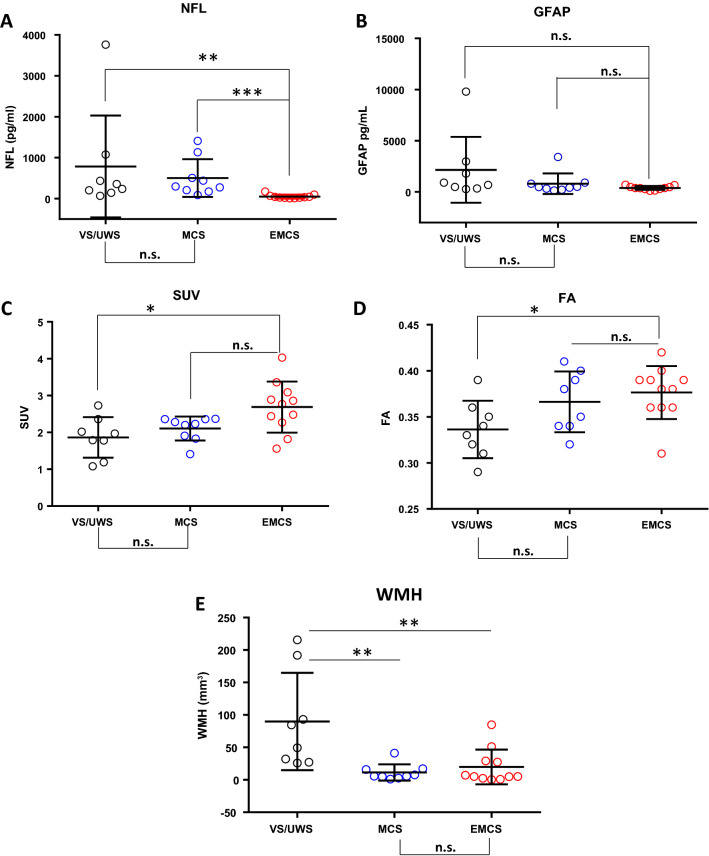
NFL (**A**), and GFAP (**B**) circulating biomarkers in pDoC patient subgroups. Whole-brain SUV**(C)**, FA (**D**) and WMH (**E**) imaging parameters obtained by PET-MRI. The values of selected markers were obtained from two independent measurements. * = *p*-value < 0.05. ** = *p*-value < 0.01. *** = *p*-value < 0. 001. One-way anova multiple comparison (Tukey’s method).n.s. = not significant. VS/UWS (Vegetative State/Unresponsive Wakefulness Syndrome). MCS (Minimally Conscious State). EMCS (Emergence from a Minimally Conscious State).

NF-L and GFAP circulating biomarkers significantly differ between patients with pDoC and HC (Supplementary Fig. 1). The serum NF-L concentration was significantly (*p* < 0.0001, unpaired t-test) higher in pDoC patients (mean value = 405.55 pg/mL ranging from 67.53 to 3763.01 pg/ml) than HC (mean 6.62 pg/mL; ranging from 4.39 to 12.81 pg/ml; Supplementary Fig. 1A). Accordingly, the GFAP in pDoC patients (mean = 1027.33 pg/mL ranging from 136.73 to 9804.40 pg/ml) was significantly (*p* < 0.0001, unpaired t test) higher compared to the HC (mean = 67.11 pg/ml ranging from 39.67 to 97.12 pg/ml; Supplementary Fig. 1B). As expected, NF-L concentration negatively correlates with TFI (Supplementary Fig. 2A), while GFAP concentration does not correlate with this parameter (Supplementary Fig. 2B). Based on CRS-R total score, we stratified the patients into three diagnostic groups (i.e., VS/UWS, MCS, EMCS), including for the sixteen patients also the data obtained during follow-up (Supplementary Fig. 3). As shown in Fig. 2A the serum NF-L level was able to significantly discriminate between subjects in VS/UWS (Cohen’s d effect size = 0.816366) or MCS (Cohen’s d effect size = 0.010614) compared to those in EMCS. It was not able to discriminate between subjects in the VS/UWS group compared to MCS, as well as for the circulating biomarker GFAP (Figs. 2B).

Using whole-brain clinical quantitative parameters derived from multimodal PET-MRI imaging analysis, the brain SUV was able to significantly discriminate only the EMCS subgroup compared to VS/UWS (*p*-value < 0.05, Cohen’s d effect size = 0.131656) (Fig. 2C). Furthermore, this parameter is in accordance with the FA parameter that was only able to significantly differentiate VS/UWS patients by EMCS patients (*p*-value < 0.05, Cohen’s d effect size = 0.133849) (Fig. 2D). Finally, the WMH significantly discriminated not only VS/UWS versus EMCS patients (*p*-value < 0.001, Cohen’s d effect size = 1.244807) but also VS/UWS versus MCS patients (*p*-value < 0.01, Cohen’s d effect size = 1.464388) (Fig. 2E). Mean brain lesion volume was 14,256 mm^3^, 443 mm^3^ and 19,946 mm^3^, for traumatic, anoxic and vascular patients, respectively. Moreover, in several cases it has not been possible to segment a focal lesion on the T1 scan, although a more diffuse altered pattern was detected by FLAIR and DWI sequences. T1-weighted axial lesion plane imaging and FLAIR for the 16 pDoC patients with VS/UWS or MCS at the time of enrollment is shown in Supplementary Fig. 4.

### Correlation of blood and imaging markers

**Figure 3:**
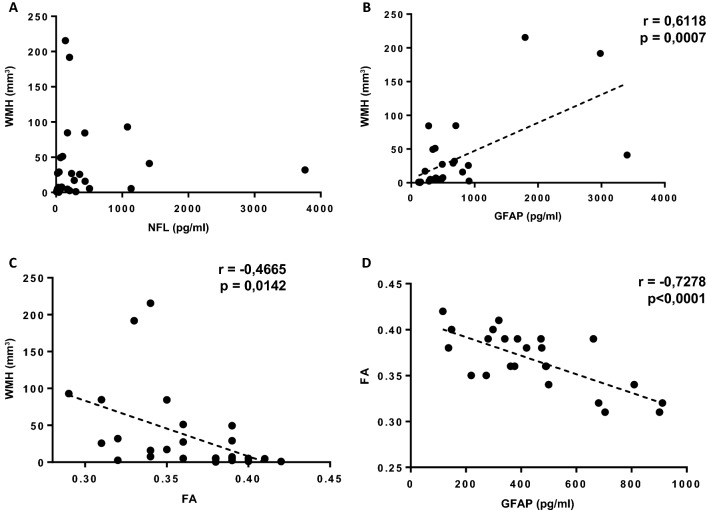
Correlation analyses between WMH vs. NF-L (**A**), WMH vs. GFAP (**B**), WMH vs. FA (**C**) and FA vs. GFAP (**D**)in pDoC patients. The values of Pearson correlation coefficients (r) and *p*- values are reported inside the graphs.

In order to highlight possible correlation and/or redundancy of selected parameters, we decided to correlate clinical imaging parameters with data obtained from the circulating blood markers. We did not observe any correlation for the lesion brain volume (mm^3^) with the circulating blood biomarkers evaluated. As shown in Fig. 3A the WMH values did not correlate with the NF-L biomarker. Conversely a direct correlation with the glial marker GFAP emerged (Fig. 3B, r = 0.6118, *p*-value = 0.007). Since, as expected, FA inversely correlates with WMH lesion volumes (Fig. 3C), we decided to evaluate the possible correlation between FA and circulating glial marker, GFAP. In this case too, a strong and significant negative correlation was detected (r = − 0.7278 and *p*-value = 0.0001) (Fig. 3D).

### pDoC markers in prognosis

**Figure 4:**
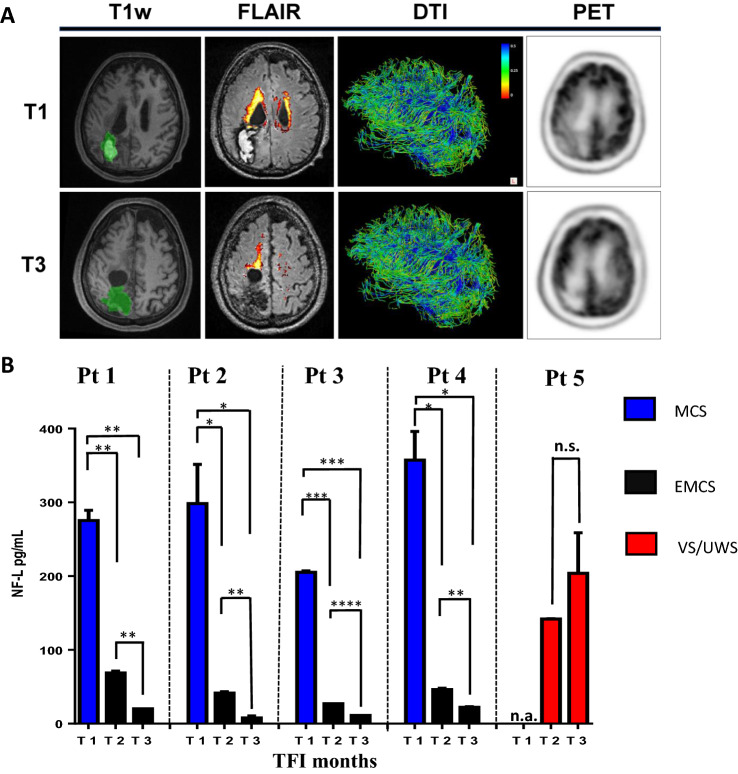
(**A**) PET/MRI scans of a 60yo patient with hemorrhagic parieto-occipital lesion and secondary enlargement of correspondent lateral ventricle, following percutaneous transluminal coronary angioplasty (T1 [VS/UWS] and T3 [EMCS], respectively at 4 and 18 months since injury). From left to right, a T1w sequence showing segmented hemorrhagic lesion (green) with blood hyperintense signal in transparence at T1; a FLAIR sequence with automatic LPA-segmentation of mostly periventricular hyperintense gliotic alterations; DTI reconstruction of streamlines encoded for scalar FA value (brain lateral view); axial view of 18F-FDG PET at both the time points. (**B**) The serum NF-L marker was evaluated in n = 5 pDoC patients at 1–6, 7–12, and 13–18 months after brain injury.* = *p*-value < 0.05.** = *p*-value < 0.01.*** = *p*-value < 0.001.**** = *p*-value < 0.0001. The values of selected markers were obtained from two independent measurements. TFI = time from injury expressed in months. n.a. = not applicable. The blue columns refer to MCS; the red ones to VS/UWS; the black ones to EMCS.

Inside our study cohort, we identified five patients who underwent multiple assessments at different time points after injury. We measured the five markers analyzed (NF-L, GFAP, whole-brain SUV, FA, and WMH) at T1 (1–6 months from injury), T2 (7–12 months from injury), T3 (13–18 months from injury) to assess the longitudinal trend at subject level. The imaging assessment provided an objective evaluation of the brain regions involved, although morpho-functional changes (Fig. 4A), do not always significantly reflect the clinical evolution between two follow-ups (T1 and T3) (Supplementary Fig. 5 and Table 2). For the 4 out of 5 analyzed patients (Pt1, Pt2, Pt3, Pt4), who started from a state of MCS, improving to EMCS, NF-L concentrations significantly changed between T1 and T2 time point, and between the T2 and T3 assessment after the injury (Fig. 4B). Moreover, higher differences were found among those patients between T2 and T3 time points, where not even the CRS-R scale was able to evaluate significant differences (see Table 2, third and fourth columns). For Pt5, a stable patient VS/UWS, we found no significant changes in the NF-L values (Fig. 4B). For the other markers, none was able to give reproducible significant differences for all subjects tested (Supplementary Fig. 5).

**Table 2:** Measurements of biological and imaging markers.

Patients	TFI	CRS-R	Biological markers mean		Imaging biomarkers		
			NF-L (pg/ml)	GFAP (pg/ml)	BRAIN SUV (mean)	WMH mm^3^	FA
# 1	1–6	12	275.37	219.02	1.83	17.088	0.35
	7–12	22	68.7	474.64	1.82	4.946	0.38
	13–18	23	20.15	298.32	2.48	5.127	0.4
# 2	1–6	11	298.35	148.13	1.41	1.088	0.4
	7–12	23	41.29	136.73	2.86	0.866	0.38
	13–18	23	7.79	116.19	2.77	0.885	0.42
# 3	1–6	10	205.11	911.77	2.2	2.638	0.32
	7–12	22	27.02	362.2	3.36	2.527	0.36
	13–18	23	11.18	280.27	3.09	2.412	0.39
# 4	1–6	5	357.22	901.66	2.02	25.79	0.31
	7–12	21	46.28	661.92	2.27	27.096	0.39
	13–18	23	22.24	490.77	2.44	27.361	0.36
# 5	1–6	–	n.d	n.d	n.d	n.d	n.d
	7–12	6	141.89	1797.50	1.53	215,593	0.34
	13–18	8	203.88	2983.48	2	191,811	0.33

Report of the biological and imaging markers mean values of the n = 5 follow up patients analyzed. TFI, Time from injury; CRS-R, Coma recovery Scale-revised; FA, Fractional anisotropy; n.d. = not determined.

## Discussion

### Current blood pDoC markers

The introduction and the use of ultra-sensitive technologies allow discriminating the infinitesimal concentrations of cerebral injury biomarkers^22,23^. Currently, due to minimal invasiveness, it is simpler to obtain a blood sample in comparison with other types of biological samples such as cerebrospinal fluid (CSF), that needs a more invasive approach. The protein S100 and neuron-specific enolase (NSE) are the two most commonly studied blood-based biomarkers of brain injury after cardiac arrest; NSE is the only blood biomarker currently recommended by guidelines for post-cardiac arrest care^24^. Unluckily, the NSE is sensitive to a technical condition of the blood sample such as hemolysis, and its prognostic ability is reduced in elderly patients and in patients with shorter cardiac arrest duration^25^.

### Innovative approaches to study pDoC

In the present study, we used an innovative digital protein detection technology, which represents a more sensitive method compared to the traditional immune-assay techniques. We found that the serum NF-L and GFAP proteins levels were higher in pDoC patients compared to the HC. Furthermore, we correlated the data obtained by the detection of serum biomarkers with imaging data obtained from simultaneous PET-MRI. The analyses of two blood biomarkers, such as NF-L and GFAP, and imaging parameters, i.e., whole-brain SUV, FA and WMH, prompted us to deepen the pDoC patients in VS/UWS, MCS and EMCS subgroups. While it is not surprising that brain volume lesions were not correlated to consciousness level, strictly depending on disorder of consciousness (DoC) etiology, it was interesting that the quantification of the WMH was the unique parameter able to distinguish between VS/UWS and MCS patients. The automatic approach chosen for WMH segmentation is a robust and validated method^26,27^applied also in a clinical context. However, mainly for this kind of patient movement artifacts can bias the automatic output and should be carefully inspected. Moreover, the correlation of GFAP with FA and WMH parameters could be useful to evaluate the assessment of the restoration of brain function, suggesting this blood marker as less-expensive imaging surrogate if not possible. Moreover, it is important to highlight the potential use of peripheral NF-L biomarker for the longitudinal assessment of the consciousness recovery, during follow-up and patient chronic management. In this scenario, there are several efforts to include in clinical practice biomarkers that can provide a quantitative value of neuronal damage and recovery of cognitive functions. Other studies have investigated the role of NF-L associated with imaging parameters; Ljungqvist J et al. (2017) found that the mean NF-L concentrations among the patients with DoC (n = 9) displayed a 30-fold increase compared to the controls, significantly differentiating these two groups; they also found a relation between serum NF-L and MR-DTI parameters, with higher NF-L concentrations in patients with higher trace (R^2^ = 0.79) and lower FA (R^2^ = 0.83)^28^. In their experimental setup, blood samples from the patients were obtained within six days post-injury; on the contrary, we analyzed blood samples at least one month post-injury (T1), correlating NF-L with FA and also analyzing GFAP as marker of astrogliosis^28^. Recently, Bagnato et al. (2021) found that the serum NF-L levels of patients with pDoC after traumatic brain injury or hypoxic-ischemic brain injuries at 1–3 months post-injury were more than 19 times higher than those of matched HC, and almost 5 times higher at 6 months post-injury. This study has adopted the conventional ELISA kit as a detection technique, less sensitive than the single-molecule array method for serum NF-L detection; however, comparisons between subpopulations were performed in small numbers of patients, which also represents our limitation^17^. Moreover, the temporal window of 6 months adopted for clinical use did not show results for one year after brain injury^17,29^.

### Limitations of the study

Regarding the limitations of the study, we know the small sample size and mixed etiology did not allow any generalization. Indeed, only 16 pDoC patients were studied for and evaluated, when possible, at subsequent timepoints. Even if, this combined cross-sectional and longitudinal approach within a retrospective framework, could represent a methodological issue, the possibility to compare for the same day clinical, structural and metabolic imaging parameters and peripheral blood biomarkers represent a pivotal track for new studies in this critical cohort of patients. Furthermore, we are aware that it is not always possible to perform an advanced imaging examination for these patients, due to the critical medical condition. From a clinical point of view, these patients are unstable and, despite the fact that the clinical assessments for the study were repeated at least three times, we do not exclude that there may have been misdiagnoses due to temporary fluctuating conditions. Regarding the NF-L marker, age is by far the most important factor influencing the determined circulating value. To minimize the influence of age, we analyzed a cohort with an age mean < 50 years^30,31^.

## Conclusions

Notwithstanding these limitations, our preliminary study could suggest a new multidisciplinary approach to adopt in the management of pDoC patients, to provide personalized and quantitative parameters to the clinician and improve pDoC patient management.

## Methods

### Study population

We analyzed for the retrospective study the serum samples of pDoC patients (n = 16) who underwent simultaneous PET-MRI and blood sampling in the same session. The pDoC patients were divided by clinical diagnosis (VS/UWS, MCS, EMCS) and subsequent serum samples and imaging analyses were also included in the evaluation of the markers. In addition, n = 6 HC were enrolled to determine the blood serum concentration of NF-L and GFAP proteins. For the final evaluation, n = 14 patients were excluded. To select the pDoC patients, the following inclusion criteria have been adopted: (1)clinical diagnosis of UWS, MCS or EMCS according to standard diagnostic criteria ^32^;(2) blood sampling/imaging session time from onset longer or equal than one month; (3) severe traumatic, vascular or anoxic brain injury. We excluded from the study patients with: (1) other pathologies independent from the acquired brain injury (e.g., psychiatric or neurodegenerative diseases); (2) not stabilized and severe general clinical conditions; (3) clinical diagnosis had changed in the week before the neuroimaging acquisition/sampling; (4) incomplete clinical data. The retrospective study followed the STROBE checklist (https://www.strobe-statement.org/checklists/).

### Standard protocol approvals, registrations, and patient consents

The study was approved by the local Ethics Committee of IRCCS Pascale (*Protocol number: 9/21 date: 16/12/21*) and performed according to the ethical principles introduced in 1964 by the Declaration of Helsinki and its later amendments. The legal guardians of the patients signed informed consent. We obtained blood samples from IRCCS SYNLAB SDN Biobank in Naples (Italy) ^33^,partner of the European network BBMRI-ERIC.

### Clinical assessment

One week before and one week after neuroimaging recording and blood sampling, all patients underwent at least 3 clinical evaluations, using the Italian version of the CRS-R^34^, to confirm stabilized clinical diagnosis of VS/UWS, MCS, or EMCS and to gather the best CRS-R total score. Patients’ consciousness level was also assessed in the imaging-blood sampling day by one skilled psychologist.

### Blood collection and ultra-sensitive determination

After thawing, serum samples were centrifuged at 10,000 g for 10 min at + 4 °C to eliminate any debris. The serum NF-L concentration was measured, as for other cohorts previously evaluated by others research groups^19,35,36^, using the commercially available Single Molecule Array NF-Light immunoassay Advantage kit; the GFAP Discovery kit was used for the GFAP determination. The biomarkers assays were performed using the Simoa SR-X instrument (Quanterix, Lexington, MA). The serum samples were diluted four-fold as suggested by the kit data sheet; calibrators (A-H) and quality controls were used to assess the concentration of the reference analytes. The limit of detection for the Simoa NFL assay was 0.0552 pg/ml and the lower limit of quantification was 0.316 pg/ml when compensated for a four-fold sample dilution; the limit of detection for the Simoa GFAP assay was 0.26 pg/ml and the lower limit of quantification was 1.37 pg/ml when compensated for a four-fold sample dilution. Samples were analyzed in duplicate, and the CV for the samples was below 20%. The researchers who performed the NF-L and GFAP assays had no information related to the clinical data. Blood samples from six HC stored in the IRCCS SYNLAB SDN Biobank were extracted and processed in the same manner.

### PET/MRI acquisition protocol

PET/MRI data were simultaneously acquired using a Biograph mMR tomograph (Siemens Healthcare, Erlangen, Germany) designed with a multi-ring LSO detector block embedded into a 3 T magnetic resonance scanner. The nominal axial and transverse resolution of the PET system was 4.4 and 4.1 mm FWHM, respectively, at 1 cm from the isocenter. Additional technical details on the scanner are reported elsewhere ^37^. A dynamic brain PET study was performed after the intravenous bolus administration of ^18^F-fluorodeoxyglucose (^18^F-FDG) tracer. PET acquisition started following the i.v. injection of 5 MBq/Kg of 18F-FDG. No food or sugar was administered to the subjects for at least 6 h prior to FDG injection. Blood glucose was measured at arrival at the PET center in all cases, and FDG was injected only if glycemia was below 120 mg/dl.

The PET data were acquired in list mode for 60 min; the matrix size was 256 × 256. PET emission data were reconstructed with ordered subset-expectation maximization (OSEM) algorithm (21 subsets, 4 iterations) and post-filtered with a three-dimensional isotropic Gaussian of 4 mm at FWHM. Attenuation correction was performed using MR-based attenuation maps derived from a dual-echo (TE = 1.23–2.46 ms) Dixon-based sequence (repetition time 3.60 ms), allowing for reconstruction of fat-only, water-only, and of fat–water images ^38^. During PET acquisition, the following MRI sequences were sequentially run: I. 3D T1-weighted magnetization-prepared rapid acquisition gradient-echo sequence (MPRAGE, 240 sagittal planes, 256 × 214 mm field of view, voxel size 0.8 × 0.8 × 0.8 mm3, TR/TE/inversion time (TI) 2400/2.25/1000 ms, flip angle 8°, acquisition time (TA) = 6′18″) II. 3D fluid attenuation inversion recovery (FLAIR, 160 sagittal planes, 192 × 192 mm field of view, voxel size 1 × 1 × 1 mm^3^, TR/TE/TI 5000/334/1800 ms, TA = 6′42″). III. Diffusion tractography (TR: 3851, TE: 84.2 voxel: 2 mm^3^ isotropic, axial planes; 71 directions; b value max: 1500, matrix: 128 × 128 acquired both with Anterior–Posterior and Posterior-Anterior phase encoding).

### Structural data processing

Anatomical Data Preprocessing Anoxic/traumatic lesion volumes (LV) were manually segmented on a 3D T1 sequence by an expert neuroradiologist, using Mimics inPrint (Materialise, Leuven, Belgium). White matter hyperintensities (WMH) are markers of white matter tissue damage seen as hyperintense signals on FLAIR (Fluid Attenuated Inversion Recovery) images. We selected an open-source tool, Lesion Prediction Algorithm (LPA), from the Statistical Parametric Mapping (SPM12) software package, which automatically segments WMH using FLAIR images^39^.Although only the LPA only requires the FLAIR, the optional T1-weigthed MPRAGE was input for improved segmentation. Moreover, LPA outputs have been visual inspected by an expert neuroradiologist (CC) and results severely biased by movement artifacts have been excluded.

All processing steps for microstructural analysis were performed with the MRTrix toolbox, version 3.0^40^. Specifically, tractography was reconstructed for each subject using a default deterministic algorithm, and mean fractional anisotropy (FA) was derived for whole-brain streamlines^14^.

### Functional data processing

PET attenuation coefficients (AC) were calculated from the dual-echo Dixon images. The SUVs were calculated automatically by the software (Syngo.via, Siemens Medical Systems) using the body-weight method: SUV = [decay corrected tissue activity (kBq/ml)]/[injected 18F-FDG dose per body weight (kBq/g)]. The mean brain SUV (SUV mean) of the whole brain was derived automatically by the software using a voxel-of-interest (VOI) method of tissue delineation.

### Statistical analysis and reproducibility

Due to small sample sizes, a non-parametric test (Kruskal–Wallis) with pairwise posthoc analysis using Bonferroni correction was used to compare the differences between groups. P-values were calculated as described in individual figure legends using Graphpad Prism 7 (Graphpad Software) and considered significant below 0.05. Cohen’s effects were calculated using the following link: https://www.socscistatistics.com/effectsize/default3.aspx. Numbers of biological and/or technical replicates as well as a description of the statistical parameters are stated in the figure legends. No statistical method was used to predetermine sample size, and experiments were not randomized.

## Supplementary Information

Supplementary Information.

## Data Availability

The datasets used and analyzed during the current study are available from the corresponding author on a reasonable request.

## Abbreviations

Brain SUV: Brain standardized uptake value
CRS-R: Coma recovery scale-revised
CSF: Cerebrospinal fluid
DoC: Disorder of consciousness
EMCS: Emergence from the minimally conscious state
FA: Fractional anisotropy
GFAP: Glial fibrillary acidic protein
MCS: Minimally conscious state
NF-L: Neurofilament light chain
NSE: Neuron-specific enolase
PDoC: Prolonged disorders of consciousness
VS/UWS: Vegetative state/unresponsive wakefulness syndrome
TFI: Time from injury
WMH: White matter hyperintensities
