# Supplementary material for: An innovative approach for the evaluation of prolonged disorders of consciousness using NF-L and GFAP biomarkers: a pivotal study
Source: Sci Rep. 2022 Nov 2;12:18446. doi: 10.1038/s41598-022-21930-w (PMC9630372; doi:10.1038/s41598-022-21930-w)
Supplement: Supplementary file 1 — Supplementary Information. [file 41598_2022_21930_MOESM1_ESM.pptx]

## Slide 1
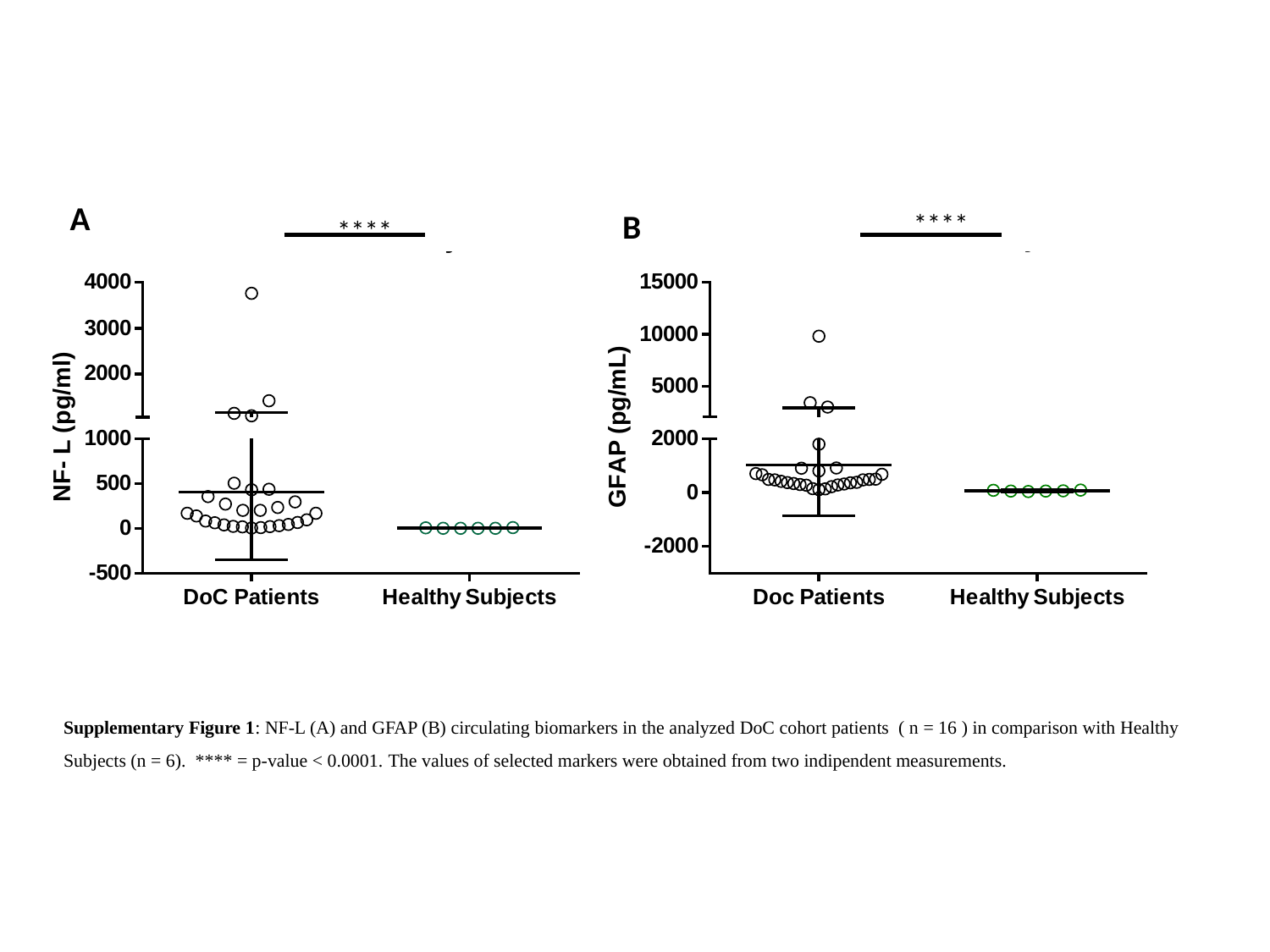

A
B
****
****
Supplementary Figure 1: NF-L (A) and GFAP (B) circulating biomarkers in the analyzed DoC cohort patients ( n = 16 ) in comparison with Healthy Subjects (n = 6). **** = p-value < 0.0001. The values of selected markers were obtained from two indipendent measurements.

## Slide 2
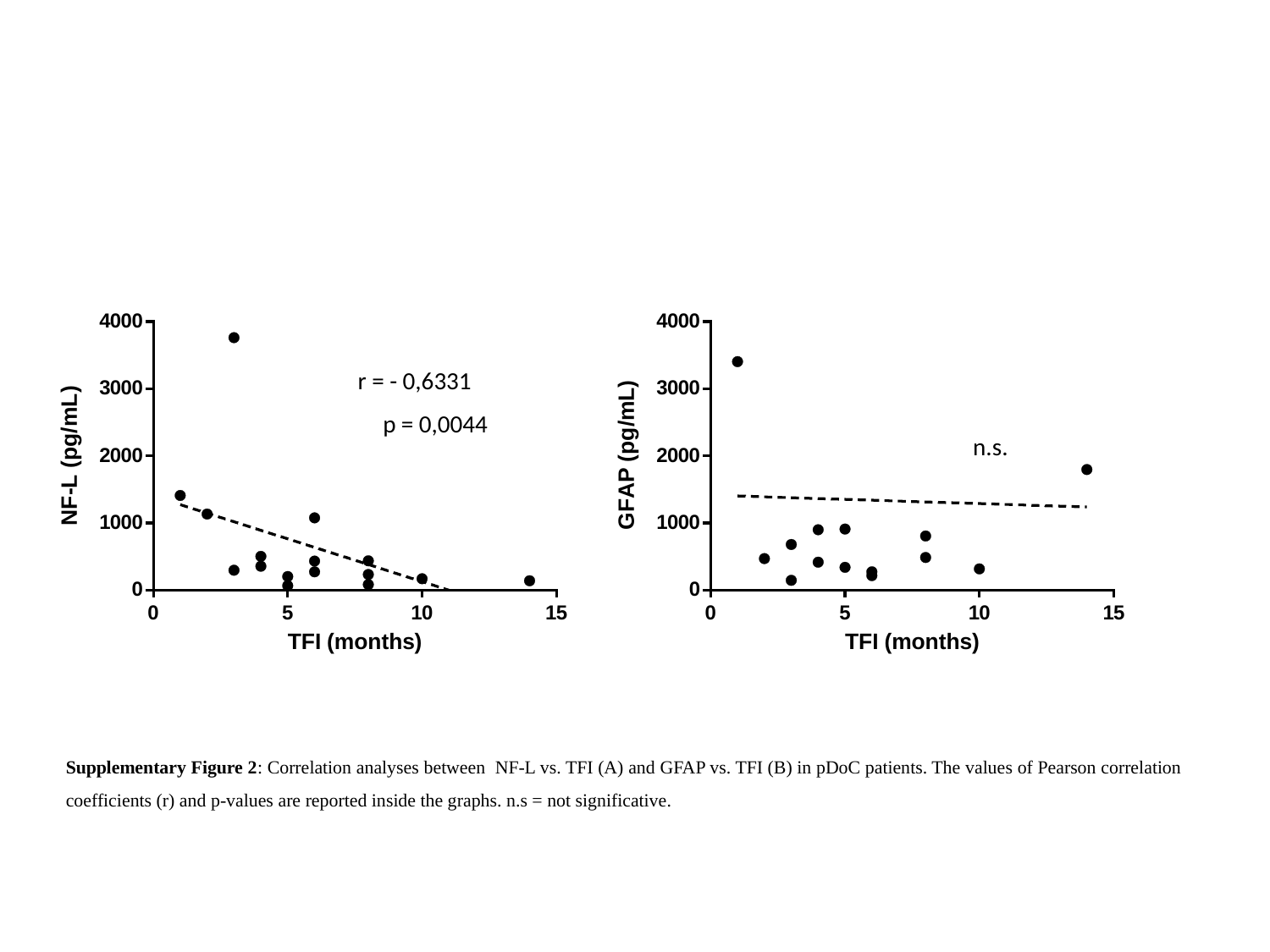

r = - 0,6331
p = 0,0044
n.s.
Supplementary Figure 2: Correlation analyses between NF-L vs. TFI (A) and GFAP vs. TFI (B) in pDoC patients. The values of Pearson correlation coefficients (r) and p-values are reported inside the graphs. n.s = not significative.

## Slide 3
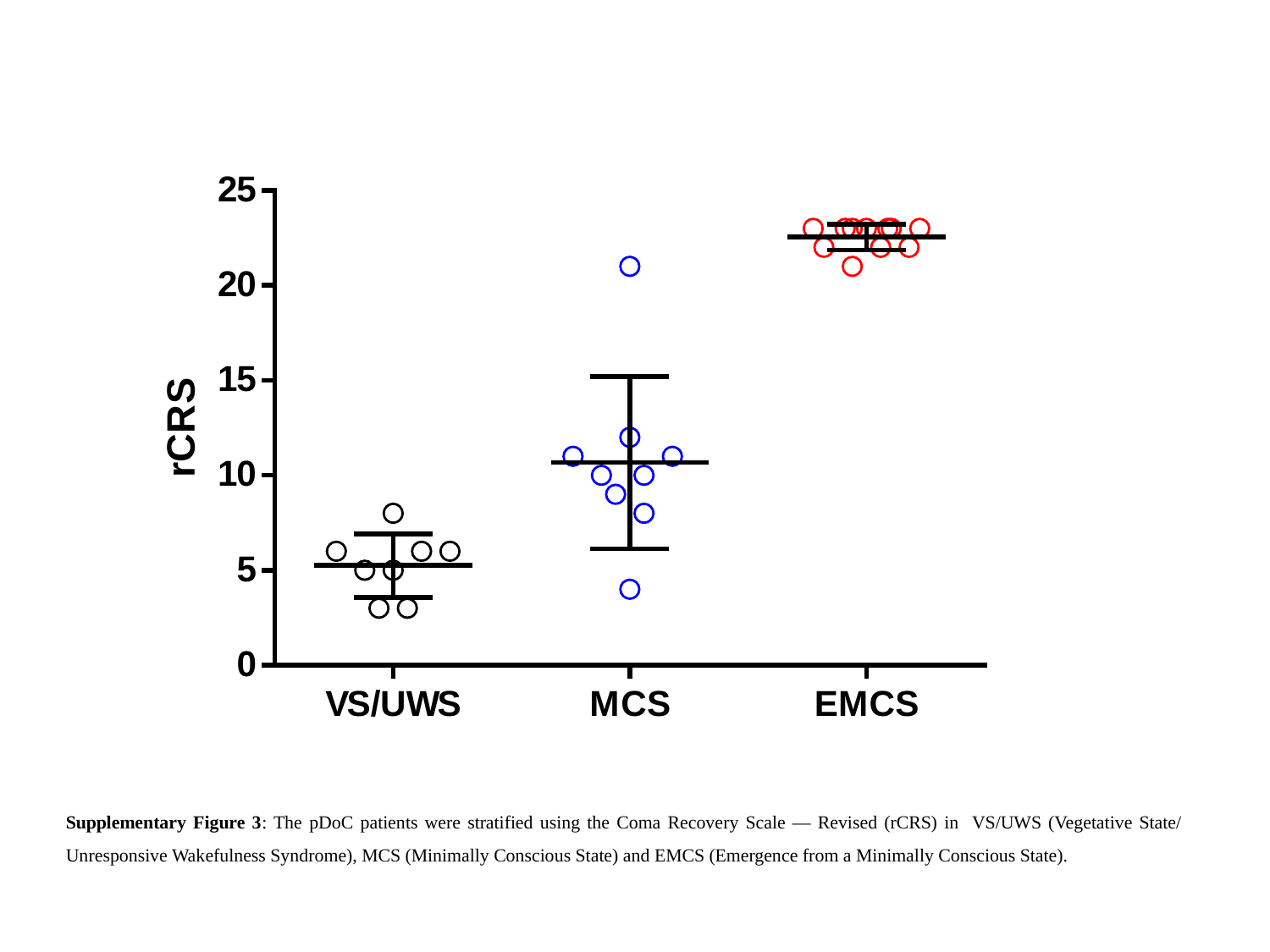

Supplementary Figure 3: The pDoC patients were stratified using the Coma Recovery Scale — Revised (rCRS) in VS/UWS (Vegetative State/ Unresponsive Wakefulness Syndrome), MCS (Minimally Conscious State) and EMCS (Emergence from a Minimally Conscious State).

## Slide 4
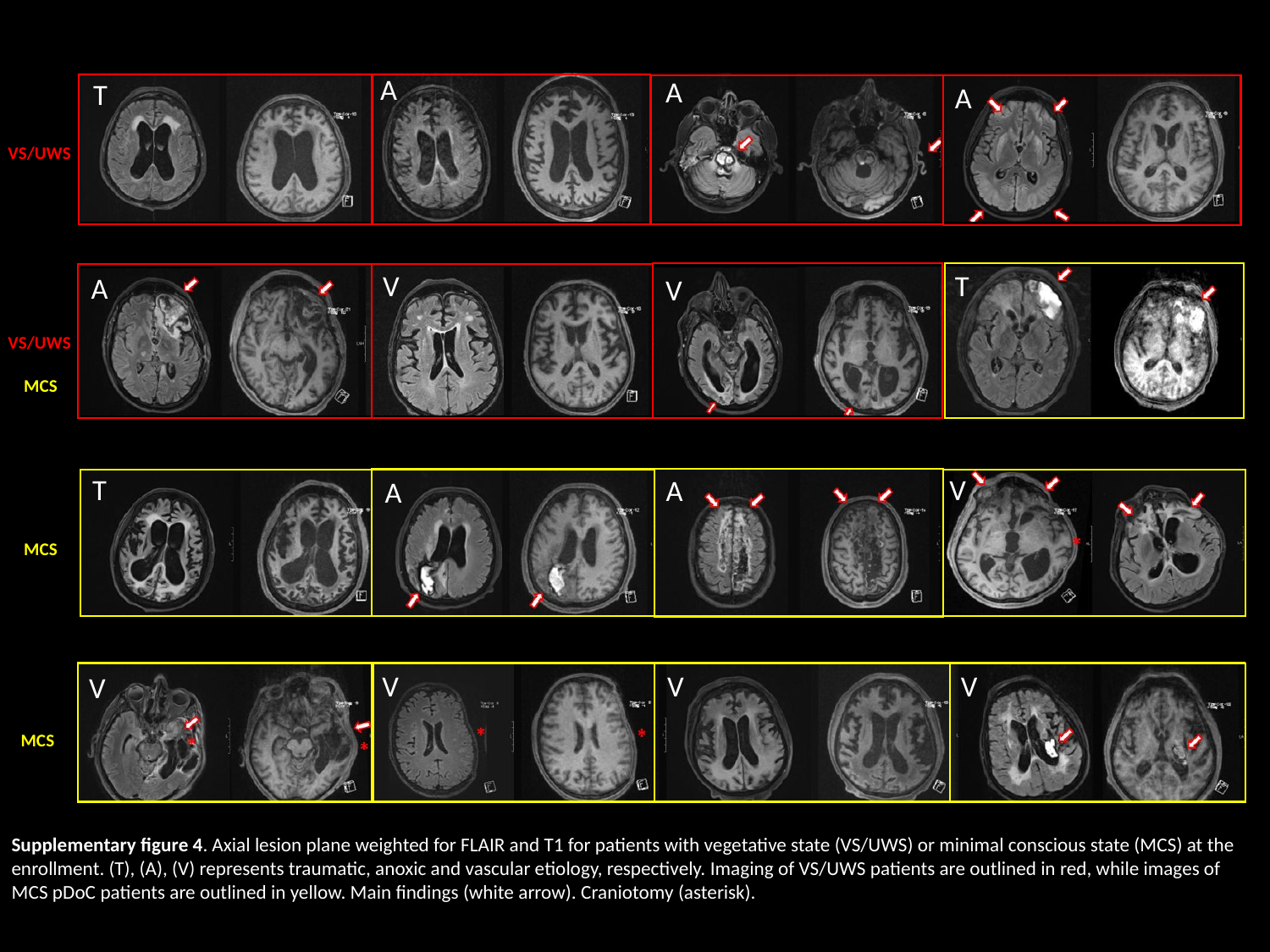

A
A
T
A
VS/UWS
T
V
A
V
VS/UWS
MCS
V
T
A
A
MCS
V
V
V
V
MCS
Supplementary figure 4. Axial lesion plane weighted for FLAIR and T1 for patients with vegetative state (VS/UWS) or minimal conscious state (MCS) at the enrollment. (T), (A), (V) represents traumatic, anoxic and vascular etiology, respectively. Imaging of VS/UWS patients are outlined in red, while images of MCS pDoC patients are outlined in yellow. Main findings (white arrow). Craniotomy (asterisk).

## Slide 5
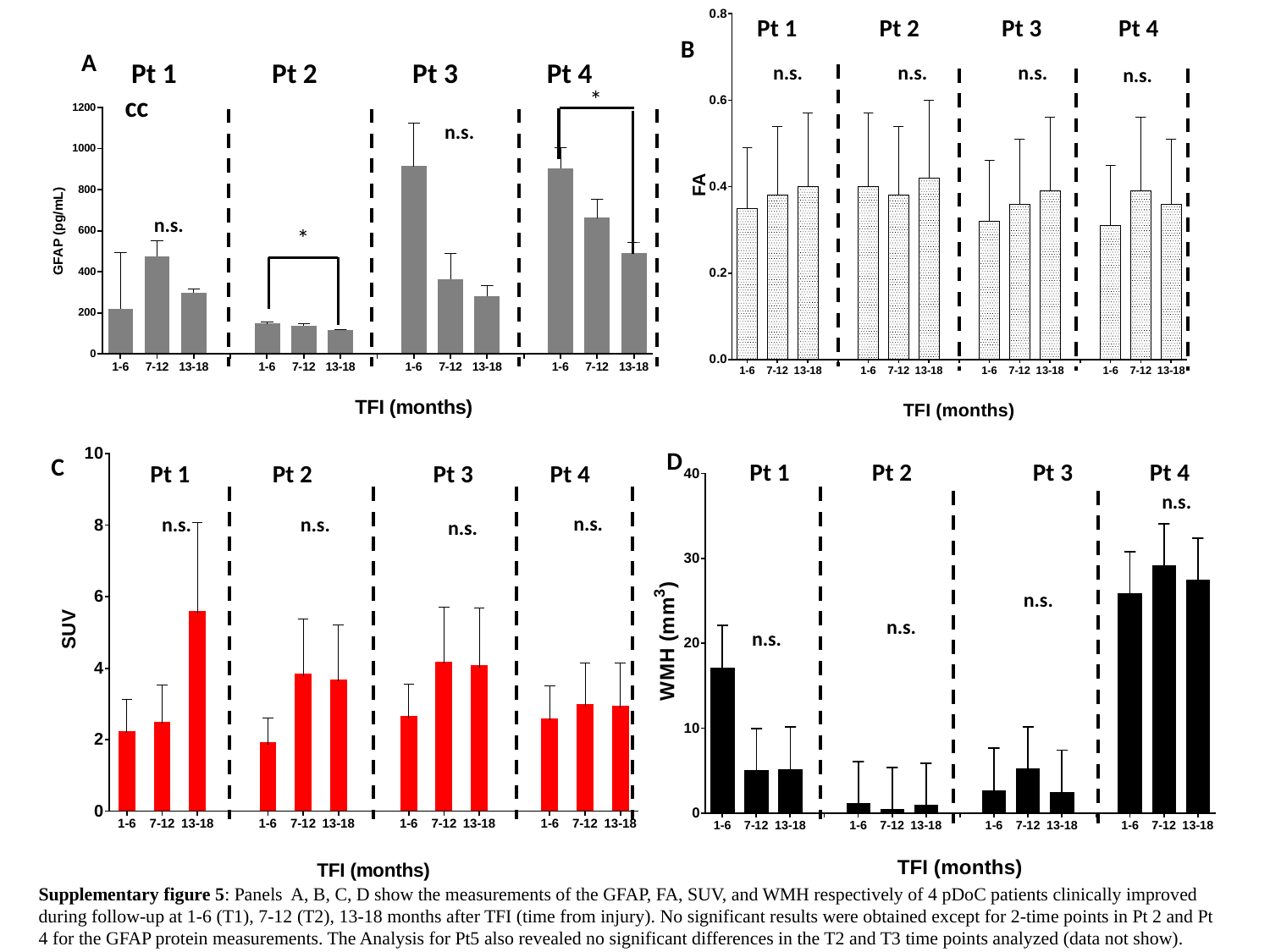

Pt 1 Pt 2 Pt 3 Pt 4
B
A
 Pt 1 Pt 2 Pt 3 Pt 4 cc
n.s.
n.s.
n.s.
n.s.
*
n.s.
n.s.
*
n.s.
n.s.
n.s.
n.s.
n.s.
n.s.
n.s.
n.s.
D
C
 Pt 1 Pt 2 Pt 3 Pt 4
 Pt 1 Pt 2 Pt 3 Pt 4
Supplementary figure 5: Panels A, B, C, D show the measurements of the GFAP, FA, SUV, and WMH respectively of 4 pDoC patients clinically improved during follow-up at 1-6 (T1), 7-12 (T2), 13-18 months after TFI (time from injury). No significant results were obtained except for 2-time points in Pt 2 and Pt 4 for the GFAP protein measurements. The Analysis for Pt5 also revealed no significant differences in the T2 and T3 time points analyzed (data not show).
